# Mr. Whately on Procidentia Ani

**Published:** 1812-04

**Authors:** Thomas Whately

**Affiliations:** Grafton-street


*To the Editors of the Medical and Physical Journal.*

GENTLEMEN,

IF you deem the following case, of a very troublesome and not unfrequent complaint, worthy a place in your valuable miscellany, it is at your service.

I am, Gentlemen,
Your obedient, humble, Servant,
THOMAS WHATELY.

*Grafton-street, Feb.* 22, 1812.

John Crew, aged 50, No. 1, Wheeler-street, Bethnal-Green, has been subject to Procidentia ani for three or four years. The gut however came down only occasionally, as, for instance, when he was at hard labor. When this happened he used to push the prolapsed part up again, and he became became easy. About four months since, the patient having carried a sack of potatoes, the gut came down in a very violent and unusual manner, protruding itself to a much greater extent than before. The patient pushed it up; but it would not continue in its situation. From that time till I saw him (a period of four months) it was constantly in a prolapsed state, night and day, to the size of an egg. He repeatedly returned it, but it always came down again immediately. During this long period, he was utterly unable to follow his employment; and for about half that time he was confined to the house, and generally to his bed, through the uneasiness and sometimes violent pain produced by the complaint, to alleviate which he was often obliged to take laudanum. The prolapsed part was of a dark red color, and had the appearance of being strangulated by the sphincter ani. An hæmorrhage, sometimes to the quantity of half a pint at a time, used to take place two or three times a week.

At the commencement of this violent attack, he was for about three weeks under the care of a surgeon, who cut the part, which bled freely, and some ease ensued. The patient, however, received no material benefit. He was then nine weeks under the care of a surgeon in a public institution, but no amendment followed. At the expiration of this period he came under my care. I returned the gut, and kept it in its situation for a short time; but it prolapsed immediately on withdrawing my fingers. He used an astringent wash, and put up the gut frequently himself; but it always came down again immediately. Finding this treatment fail, I directed him to pass, beyond the sphincter ani, a bougie well oiled, about the size of a middle-sized finger, and four inches long, with a piece of fine tape fastened in a strong manner to one extremity of it, by boring a hole through its substance. This was buried in its situation daily for about two hours. It had the effect of keeping up the rectum during the time it remained; but on withdrawing it, the complaint became as bad as ever, and at the end of a week he was very little better. I then directed him to pass in the same manner, and for the same length of time, another bougie of the length of the former, but about three quarters of an inch in diameter. This he did five times in the course of a week, but, on withdrawing it, the procidentia always returned immediately, and he appeared to be no better. He then kept it in the same position during a whole night, and withdrew it in the morning by the string. This method has succeeded. He is now so well by only once introducing the bougie in this manner, as to be able to follow a laborious employment.

employment. A very small part of the gut (not larger than a nut) still generally comes down when he goes to stool; but he returns it readily by a very slight pressure, and it remains in its proper situation. The same slight appearance of the complaint likewise sometimes takes place when he speaks aloud; but a trifling pressure remedies the inconvenience. It is scarcely necessary to observe, that the rectum will take a much larger bougie than what I used in this case.

